# Risk factors for colonization with extended-spectrum cephalosporin-resistant and carbapenem-resistant Enterobacterales among hospitalized patients in Guatemala: An Antibiotic Resistance in Communities and Hospitals (ARCH) study

**DOI:** 10.1016/j.ijregi.2024.100361

**Published:** 2024-03-30

**Authors:** Mark A. Caudell, Carmen Castillo, Lucas F. Santos, Laura Grajeda, Juan Carlos Romero, Maria Renee Lopez, Sylvia Omulo, Mariangeli Freitas Ning, Guy H. Palmer, Douglas R. Call, Celia Cordon-Rosales, Rachel M. Smith, Carolyn T.A. Herzig, Ashley Styczynski, Brooke M. Ramay

**Affiliations:** 1 Washington State University, Paul G. Allen School for Global Health, Pullman, USA; 2 Universidad del Valle de Guatemala, Center for Health Studies, Guatemala City, Guatemala; 3 Washington State University, Global Health-Kenya, Nairobi, Kenya; 4 U.S. Centers for Disease Control and Prevention, Guatemala City, Central America Regional Office, Guatemala; 5 U.S. Centers for Disease Control and Prevention, Division of Healthcare Quality Promotion, Atlanta, USA

**Keywords:** Antimicrobial resistance, Colonization, Hospitals, Guatemala

## Abstract

**Objectives:**

The spread of extended-spectrum cephalosporin-resistant Enterobacterales (ESCrE) and carbapenem-resistant Enterobacterales (CRE) has resulted in increased morbidity, mortality, and health care costs worldwide. To identify the factors associated with ESCrE and CRE colonization within hospitals, we enrolled hospitalized patients at a regional hospital located in Guatemala.

**Methods:**

Stool samples were collected from randomly selected patients using a cross-sectional study design (March-September, 2021), and samples were tested for the presence of ESCrE and CRE. Hospital-based and household variables were examined for associations with ESCrE and CRE colonization using lasso regression models, clustered by ward (n = 21).

**Results:**

A total of 641 patients were enrolled, of whom complete data sets were available for 593. Colonization with ESCrE (72.3%, n = 429/593) was negatively associated with carbapenem administration (odds ratio [OR] 0.21, 95% confidence interval [CI] 0.11-0.42) and positively associated with ceftriaxone administration (OR 1.61, 95% CI 1.02-2.53), as was reported hospital admission within 30 days of the current hospitalization (OR 2.84, 95% CI 1.19-6.80). Colonization with CRE (34.6%, n = 205 of 593) was associated with carbapenem administration (OR 2.62, 95% CI 1.39-4.97), reported previous hospital admission within 30 days of current hospitalization (OR 2.58, 95% CI 1.17-5.72), hospitalization in wards with more patients (OR 1.05, 95% CI 1.02-1.08), hospitalization for ≥4 days (OR 3.07, 95% CI 1.72-5.46), and intubation (OR 2.51, 95% CI 1.13-5.59). No household-based variables were associated with ESCrE or CRE colonization in hospitalized patients.

**Conclusion:**

The hospital-based risk factors identified in this study are similar to what has been reported for risk of health care–associated infections, consistent with colonization being driven by hospital settings rather than community factors. This also suggests that colonization with ESCrE and CRE could be a useful metric to evaluate the efficacy of infection and prevention control programs in clinics and hospitals.

## Highlights

• Among inpatients, human colonization with cephalosporin- and carbapenem-resistant bacteria was high.
• Cephalosporin and carbapenem administration were predictive of colonization.
• Length of stay, previous hospitalization, and intubation were also predictive.
• No household-level variables predicted colonization in the hospital.
• Colonization may serve as a useful metric to assess infection prevention and control programs in hospitals.

## Introduction

Hospitals administer large quantities of diverse antibiotics and are considered hotspots for the emergence and spread of antimicrobial-resistant organisms. The administration of broad-spectrum antibiotics, for example, increases the selection for multidrug-resistant (MDR) organisms [1,2], thereby increasing the abundance of these organisms and consequently the probability of transmission to patients. Emergence of hospital-acquired MDR infections results in prolonged hospital stays and increased morbidity and mortality [3]. During the COVID-19 pandemic, for example, it was estimated that over 40% of the 24,900 people who succumbed to antibiotic-resistant infections associated with health care in the United States acquired these infections within a hospital [4]. Colonization with MDR organisms can lead to infection and/or transmission to other hospitalized patients via the hospital environment or health care staff [5,6]. Discharged patients carrying MDR organisms can transmit these bacteria to household members and surrounding communities, a process thought to promote the emergence of community-acquired resistant infections, such as infections caused by bacteria that produce extended-spectrum β-lactamases [7].

Research in hospitals has consistently identified positive associations between antibiotic use and infection or colonization with MDR bacteria [8], [9], [10]. Additional correlates of MDR infection or colonization within clinical settings include measures of health care exposure such as invasive procedures, use of catheters or intubation [11], [12], [13], previous hospitalization [14], [15], [16], and length of hospital stay [9,17]. However, more studies have examined the risk factors associated with infection without considering colonization with MDR organisms as an end point of interest, and most of these studies have been carried out in high-income settings. Consequently, identifying potential risk factors for MDR colonization represents an important opportunity to design proactive strategies to prevent the morbidity and mortality from MDR infections and spread of these organisms in hospitals and in communities, particularly, in settings from low- and middle-income countries.

Colonization with extended-spectrum cephalosporin-resistant Enterobacterales (ESCrE) or carbapenem-resistant Enterobacterales (CRE) predisposes patients to infection with these organisms, which can result in increased morbidity and mortality in hospitalized patients [18,19]. Beyond antibiotic administration, less is known about other factors that could contribute to colonization before or during hospitalization. Earlier work showed that the prevalence of ESCrE and CRE colonization within a regional hospital in Guatemala (67% and 37%, respectively) is relatively high compared with other low- and middle-income countries [20], [21], [22] and very high compared with high-income countries [23,24]. This high prevalence offers an opportunity to detect potential contributions from the community to colonization in hospitalized patients.

## Methods

This cross-sectional study was carried out in the Western Highlands of Guatemala in the department of Quetzaltenango, where both Spanish-speaking Latino and Mam- and Quiche-speaking Indigenous populations reside in urban and rural populations. This region is characterized by marked variation in sanitation, exposure to animals (both through ownership of and consumption of livestock), and local access to health care [25]. Residents from 19 municipalities are served by a single tertiary referral hospital, Hospital Regional San Juan de Dios de Occidente in Quetzaltenango, that served as the study site (253 adult beds, 49 pediatric beds, and 118 infant beds, serving a population of 180,000).

### Hospital data collection

A list of eligible patient wards was generated at the study outset and visited in random order from March to September 2021 to recruit and enroll eligible participants of all ages. During enrollment, the number of occupied beds was determined by carrying out a census, including any patient admitted to a ward by 8 AM of the day that the project team arrived. In wards with ≤15 patients, all willing patients were recruited. If >15 patients were present, patient enrollment included random selection of up to 15 patients. Written informed consent was carried out in Spanish or Mam by bilingual nurses. Outpatient services/wards and COVID-19 wards were excluded from the sampling frame. In addition, patients were excluded (i) if they had documented severe neutropenia, defined as absolute neutrophil count <1700 cells/µl for people aged ≤12 years or <1000 cells/µl for people aged >12 years, (ii) if they had gastrointestinal bleeding, or (iii) if they had COVID-19. The study protocol was approved by the Universidad del Valle de Guatemala (UVG) Center for Health Studies Ethics committee (202-10-2019) and the Guatemalan Ministry of Health Ethics Committee (49-2019).

Information from patient medical records was collected to identify hospital-based risk factors potentially associated with ESCrE or CRE colonization, including diagnosis upon admission, days of hospitalization, ward(s) occupied during hospitalization, history of oral and intravenous antibiotic use, invasive and noninvasive procedures, use of medical devices, and infectious disease diagnoses during hospitalization. Questionnaire data were collected from participants or parents or guardians to identify the potential risk factors that were unavailable in medical charts but might be associated with MDR colonization (e.g. information about previous hospitalizations, antibiotic use before admission, and history of chronic diseases). Household-level variables were also collected, including information about infrastructure, sanitation, hygiene, and household assets. Collected asset data, based on the demographic health survey list [26], included having electric power and whether the household owned solar panels, radio, television, mobile/landline phones, refrigerator, washing machine, clothes dryer, microwave, computer, internet, watches, bicycle, motorcycle, and car. Also collected were details of the patient's medical history before admission, including clinic visits, hospitalizations, antibiotic use, and history of illnesses with a potential impact on immune response.

### Laboratory procedures

Detailed laboratory methods are described elsewhere [22]. Briefly, stool samples were collected in stool cups and transported at 4°C to the laboratory where swabs (FLOQSwabs) were used to transfer subsamples into eSwab liquid Amies medium, supplemented with sterile glycerol (20% v/v) and were transported with dry ice to a UVG central campus laboratory for processing. Stool sample swabs were thawed and cultured on CHROMagar extended-spectrum β-lactamases (for the detection of ESCrE) and on CHROMagar mSuperCarba (for the detection of CRE). Agar was reconstituted at the laboratory and every batch was tested with manufacturer-recommended reference strains.

Plates were incubated at 37°C for 24 hours. For each stool sample, up to three morphologically distinct colonies were selected, sub-cultured, and stored with 20% glycerol in sterile 1 × phosphate-buffered saline solution (−80°C) until shipment to the laboratory at Washington State University. The isolates were then cultured on tryptic soy agar, followed by testing using VITEK 2 GN ID and AST-GN84 reagent cards to characterize Gram-negative bacteria identity and antibiotic susceptibility profiles, respectively. Manufacturer-recommended reference strains were used to test cards and for instrument validation. Break points for antibiotic susceptibility classifications followed the guidelines from the Clinical and Laboratory Standards Institute M100-S31 [27].

### Data management and analysis

Questionnaire, medical chart review, sample collection date, and isolate data were entered into a REDCap database stored on a secure server. Isolates identified as Enterobacterales were considered ESCrE if they were resistant to ceftriaxone and susceptible or intermediate to all carbapenem antibiotics tested (ertapenem, imipenem, and meropenem). Enterobacterales isolates were classified as CRE if they exhibited resistance to one or more of the tested carbapenems. Participants were classified as being colonized with ESCrE or CRE if at least one isolate obtained from a single swab of their stool sample met these definitions.

Lasso logistic regression models were used to examine associations between multiple variables and ESCrE or CRE colonization [28]. Lasso estimation is used to regress a set of control variables on the outcome of interest (colonization) and a set of retained independent variables that are hypothesized to be associated with ESCrE or CRE colonization. The set of control variables can be large (i.e. even more than the number of observations in the data set) because lasso estimation serves as a variable reduction technique. The Supplement contains a further description of variable reduction process in lasso. Lasso logistic regression models were run using the *xplogit* command in Stata v.17 using the cross-fit partialing-out method (or double machine learning) to identify the correlates associated with ESCrE or CRE colonization and to select which control variables should be retained in the model. The *xplogit* process runs the lasso estimation across a series of data sets resampled from the original data set, 10-fold in our case, using estimates from previous runs to train subsequent estimates [29]. The calculation of interclass correlations revealed the presence of clustering by the ward in which the patients were sampled with the interclass correlations for CRE being 12% and for ESCrE being 1%. Because of the presence of clustering, a clustered sandwich estimator (clustered at the ward level) was used to estimate the variance-covariance matrix. Model results are presented as odds ratios (ORs). Data were cleaned and analyzed using Stata v.17 [30].

Independent variables known to be associated with colonization and or infection with antibiotic-resistant bacteria based on the literature were retained in the models. These variables included carbapenem or ceftriaxone administration between the time of admission and sample collection number of other patients in a ward (a continuous variable), a binary variable indicating whether the patient was intubated during hospitalization, length of hospital stay before sample collection (<4 days vs ≥4 days), and a categorical variable indicating the number of invasive procedures performed (zero, one, two or more). Procedures were classified as invasive based on the study by Sian et al. [31] (The Supplement contains more details; intubation is classified separately from other invasive procedures).

Models included a set of continuous and categorical control variables that, if deemed relevant by lasso estimation (i.e. *P* <0.05), could be retained and influence model estimation, but were otherwise removed. Four continuous variables were evaluated for inclusion in the model (patient age, wealth score [Supplement contains more information on wealth score construction], number of animals kept, including food and companion animals, and number of antibiotics received during hospitalization). Categorical variables (n = 119) included antibiotic use within 30 days before admission, admittance factors (e.g. reasons for admission, admission with infectious disease, etc.), water, hygiene and sanitation variables, indicators of health-seeking practices (e.g. visiting a clinic), items from a standard household inventory, animal ownership, ethnic and linguistic background, sex, and relevant family member activities (e.g. if a family member visited the hospital or worked in the market, etc.). The entire set of control variables used, along with definitions and descriptive statistics, are found in Supplementary Tables 1-2.

## Results

Samples, chart, and questionnaire data were collected from 641 patients. A total of 45% of all enrolled participants were adults, with a median age of 42 years and a range of 18-86 years. The median age of hospitalized children (aged ≥1 and ≤17 years) was 4 years, whereas hospitalized infants aged between 1 and 11 months had a median age of 3 months. Most patients (68%) were hospitalized for 4 or more days at the time of enrollment (median = 5 days, interquartile range 3-9 days).

The prevalence of ESCrE and CRE colonization for the entire population was 72.3% and 34.6%, respectively. The distribution of ESCrE and CRE by age groups is published elsewhere [22]. The frequency of ceftriaxone and carbapenem administration among all patients was 22% and 11%, respectively. Fecal colonization prevalence and antibiotic administration varied based on the time of enrollment relative to the time of admission. For example, among the 30 adult patients for whom stool samples were collected within 48 hours of hospital admission, the prevalence of ESCrE and CRE colonization was 76% and 20%, respectively, whereas after 48 hours (n = 258 adults), the prevalence was 70% and 40%, respectively. Of hospitalized adults enrolled within 48 hours, 73% (22 of 30) were administered antibiotics and, in total, 63 antibiotic administration events were recorded including ceftriaxone (11%, seven of 63 antibiotic events) and meropenem (2%, one of 63 antibiotic events). Of the 258 adult patients enrolled after 48 hours of hospitalization, 80% (206 of 258) were administered antibiotics for a total of 394 antibiotic administration events, including ceftriaxone (21%, n = 83 of 394) and meropenem (12%, n = 46 of 394).

Table 1 provides the summary statistics for independent variables included in the lasso regression models by whether the patient was colonized by ESCrE or CRE.

**Table 1:** Summary statistics for independent variables included in the lasso logistic regression models, Guatemala 2021.

Variable	ESCrE colonized Freq (%)	CRE colonized Freq (%)				
Carbapenem administered to patient	29/428 (6.8)	47/205 (22.9)				
Ceftriaxone administered to patient	104/428 (24.3)	54/205 (26.3)				
Hospitalized 4 days or more	304/428 (71.0)	180/205 (87.8)				
Hospitalized in month prior to current hospitalization	45/426 (10.6)	32/205 (15.7)				
Intubated during hospitalization	35/429 (8.2)	38/205 (18.5)				
Invasive procedures during hospitalization						
0	116/429 (27.0)	33/205 (16.1)				
1	200/429 (46.6)	99/205 (48.3)				
≥2	113/429 (26.3)	73/205(35.6)				

Variable	Median	IQR	Min-Max	Median	IQR	Min-Max
Number of other patients in ward at time of sampling	9	5-14	1-27	12	6-18	1-27

CRE, carbapenem-resistant Enterobacterales; ESCrE, Enterobacterales; IQR, interquartile range.

Outcome variables are colonization with ESCrE or CRE. The full list of control variable information is available in the supplementary material (Supplementary Tables 1-2). Not all columns add to the total sample size of 429 and 205, respectively, because of missing data. See Methods for full definitions of independent variables.

### Factors associated with Enterobacterales and carbapenem-resistant Enterobacterales colonization

The administration of carbapenems was associated with a 0.21-fold (95% confidence interval [CI] 0.11-0.42) decrease in the odds of ESCrE colonization when other independent variables were held constant (Table 2). In contrast, the administration of ceftriaxone was associated with a 1.61-fold (95% CI 1.02-2.53) increase in odds of ESCrE colonization. Hospitalization in the previous 30 days was associated with a 2.84-fold (95% CI 1.19-6.80) increase in odds of colonization. The probability of colonization with ESCrE was not associated with the number of other patients in wards, length of hospital stay, or whether a patient was intubated or received invasive procedures. The control variables retained during lasso estimation for ESCrE models are provided in Supplementary Tables 3-11.

**Table 2:** Factors associated with ESCrE and CRE colonization (n = 593 patients), Guatemala 2021.

Independent variable	ESCrE (n = 593)	CRE (n = 593)
Carbapenem administered to patient	0.21^b^	2.62^c^
	(0.11-0.42)	(1.39-4.97)
Ceftriaxone administered to patient	1.61^d^	1.19
	(1.02-2.53)	(0.75-1.89)
Number of other patients in ward at time of enrollment	1.01	1.05^c^
	(0.99-1.05)	(1.02-1.08)
Hospitalized 4 days or more at time of enrollment (1 = Yes, 0 = No)	1.40	3.07^b^
	(0.92-2.15)	(1.72-5.46)
Participant hospitalized in past month, prior to enrollment (1 = Yes, 0 = No)	2.84^d^	2.58^d^
	(1.19-6.80)	(1.17-5.72)
Patient intubated during study participation (1 = Yes, 0 = No)	0.75	2.51^d^
	(0.37-1.49)	(1.13-5.59)
Patient received one invasive procedure^a^ (1 = Yes, 0 = No)	1.22	1.25
	(0.81-1.86)	(0.74-2.11)
Patient received ≥2 invasive procedures (1 = Yes, 0 = No)	1.08	1.04
	(0.61-1.90)	(0.561.95)

Results are shown as odds ratios, followed by 95% confidence intervals. Note that the total number of observations included (n = 593) differs from the total number of participants providing samples (n = 641) due to missing data.

CRE, carbapenem-resistant Enterobacterales; ESCrE, Enterobacterales.

a Procedures were classified as invasive based on Sian et al. [31] (see more details in Supplement; note that intubation is not classified as an invasive procedure).

b *P* < 0.001.

c *P* < 0.01.

d *P* < 0.05.

**Figure 1:**
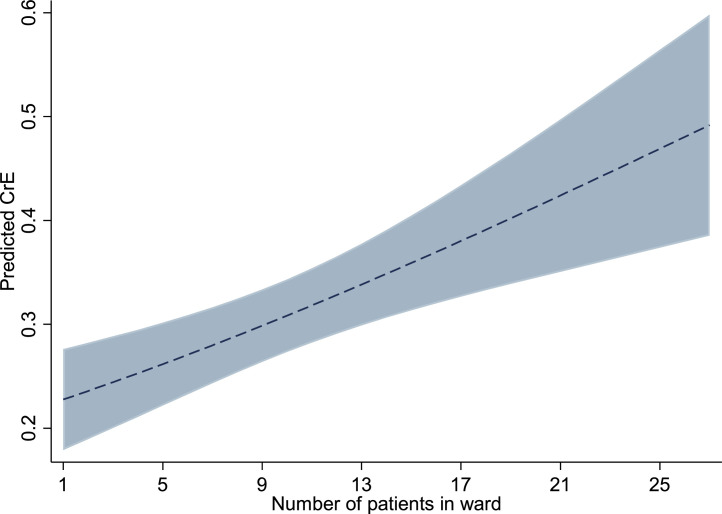
The impact of additional patients within a ward on likelihood of CRE colonization, Guatemala 2021.

For CRE, the administration of carbapenems increased the odds of colonization by 2.62 times (95% CI 1.39-4.97) when holding other independent variables constant (Table 2). For each additional patient in a respective respondent's ward, the risk of CRE colonization increased 1.05 times (95% CI 1.02-1.08). The impact of increasing the number of patients within a ward on the likelihood of being colonized by CRE, holding other correlates at mean (constant) levels, is plotted from the minimum (n = 1) to maximum (n = 27) number of other patients in a ward (Figure 1). Patients hospitalized at least 4 days before enrollment were three times more likely to be colonized with CRE (OR 3.07, 95% CI 1.72-5.46), whereas hospitalization in the previous 30 days was associated with a 2.58-fold increase in the odds of CRE colonization (OR 2.58, 95% CI 1.17-5.72). Patients who were intubated during hospitalization had a 2.51-fold (95% CI 1.13-5.59) increase in odds of CRE colonization. Ceftriaxone use and invasive procedures other than intubation were not significantly associated with CRE colonization.

CRE, carbapenem-resistant Enterobacterales.

Few covariates (Supplementary Tables 1-2) and no household-based variables were retained in the lasso models presented herein, including ownership of animals, household wealth, and water, sanitation, and hygiene factors. The iterative process of lasso modeling led to the retention of several variables, primarily hospital-based, that were associated with the main correlates. Whether a patient was admitted with an infectious disease was retained to refine the estimates between the administration of ceftriaxone and ESCrE and CRE. Admission with an infectious disease was also retained to refine the estimates between intubation during hospitalization and ESCrE. Hospital diet type (i.e.. 1200-calorie diet) was retained to refine the estimates between the administration of ceftriaxone and ESCrE. The sole household variable retained in the estimation was clinic visits (two or more times), which was retained to refine the estimates between two or more invasive procedures and ESCrE, although it was only retained in one of the 10 folds. All variables retained during lasso estimation across the 10 folds for CRE are provided in Supplementary Tables 1-2.

## Discussion

Identifying variables that are associated with antibiotic resistance within hospitals is a critical step for designing interventions to limit the health and economic burdens attributable to this challenge. These burdens are especially high for MDR infections, which are associated with higher rates of patient morbidity and mortality, prolonged hospital stays, and increased antibiotic consumption [3,32]. Data from the current analysis show that 72.3% and 34.6% of hospital patients were colonized with ESCrE and CRE, respectively, of which 83% and 100% of bacterial isolates, respectively, were resistant to three or more classes of antibiotics, thus meeting the definition of MDR organisms [33]. Monitoring colonization is also important because persons colonized with resistant bacteria are more likely to develop subsequent resistant infections [34,35], with a commensurately higher probability of readmittance. Identifying risk factors for colonization also allows potential intervention to prevent colonization and upstream of development of MDR infections.

Herein, we show that factors associated with ESCrE and CRE colonization were consistent with those documented in previous colonization studies, including antibiotic use, length of stay, previous hospitalization, and intubation [8,14]. These factors are similar to those associated with infection with antibiotic-resistant pathogens [16]. Documenting overlapping variables is important to show that robust infection prevention and control (IPC) activities may reduce colonization and infection within clinical settings. Changes in colonization status during hospitalization can be used as a key indicator when evaluating the efficacy of IPC programs. Indeed, several studies have documented the likely spread of hospital-acquired bacteria into communities [7,36]. Not only are persons who are colonized more likely to develop resistant infections, they may also contribute to subsequent spread to the broader hospital and community. Similarities between CRE strains from hospitals and communities are consistent with potential transmission between recently discharged hospitalized patients and community members [36].

Consistent with other studies in health care environments [8,14], we found that colonization with CRE is associated with previous hospitalization and length of hospital stay (i.e. 4 or more days), the latter being more predictive than carbapenem use (OR 3.07 vs 2.62, respectively). Interestingly, these similarities were evident even though the present study results were based on a random selection of patients and colonization, whereas the work in Brazil and Spain was based on clinical isolates without random selection. ESCrE colonization was also associated with previous hospitalization and was a stronger predictor of ESCrE colonization than ceftriaxone use (OR 2.84 vs 1.61, respectively). The impacts of previous hospitalization and admittance length before enrollment were maintained after controlling for antibiotic use, which highlights the importance of identifying other variables from the patient's treatment or hospital environment that could promote colonization. For example, and consistent with other studies, we show that intubation is significantly related to CRE colonization (a 2.5-fold increase in this study). In addition, although not reported frequently, we identified an association between CRE colonization and the number of other patients within wards (Figure 1), which is consistent with an increasing risk of hospital-based transmission with a higher patient density. These findings highlight the importance of implementing effective IPC activities to prevent health care–associated transmission of antibiotic-resistant organisms.

In contrast to CRE, colonization with ESCrE was not associated with any factors of a patient's current hospital stay beyond the expected positive association with ceftriaxone use and negative association with carbapenem use (ceftriaxone selects for ESCrE and carbapenems likely suppress ESCrE transmission or detection). Previous work with this same study population documented frequent use of broad-spectrum β-lactam antibiotics, including third-generation cephalosporins and carbapenems [22]. The strongest correlate of ESCrE colonization was hospitalization 30 days before study hospitalization but length of stay was not. One potential explanation for this correlation is that antibiotic use during the previous hospitalization led to a higher probability of ESCrE colonization (consistent with Table 2); however, our methods did not adequately document this previous hospital-based antibiotic selection pressure. The non-significant association between length of stay and ESCrE colonization may indicate that a high baseline saturation of community colonization (46% [22]), especially among previously hospitalized patients, does not further increase the likelihood of ESCrE colonization through transmission events occurring as hospital length of stay increases; however, antibiotic use in the hospital (e.g. ceftriaxone) occurs with sufficient frequency to select for ESCrE colonization in this population, resulting in a higher overall colonization prevalence during hospital stays.

Although our analysis controlled for numerous household-based variables that may impact transmission, including measures of water, hygiene, and sanitation, none of these variables were retained in the lasso estimation process. It is possible that household-based factors only exhibit significance when the analysis is confined to individuals recently admitted to the hospital. Although the sample size is not large enough to address this question, it is likely that after an individual has spent multiple days within the hospital, factors associated with the hospital stay, such as antibiotic use and health care–associated transmission may overwhelm household-based influences. Notably, for patients sampled within 48 hours of hospital admission, the prevalence of ESCrE and CRE colonization were 76% and 20%, respectively, at the time of sampling, which are well above the prevalence identified in the community using identical laboratory methods in within the same period (46% and 1%, respectively) [22]. This could happen if transmission and/or selection pressure begins very quickly upon admission to the hospital or if individuals admitted to the hospital are disproportionately likely to be colonized with ESCrE and CRE before admission because of history of antibiotic use, underlying diseases, or previous health care contact. In support of the former, of the 30 individuals sampled within the first 48 hours of admission, 73% had a documented administration of IV antibiotics before stool sample collection which is are likely to selectively favor ESCrE or CRE bacteria (e.g. administration of ampicillin/sulbactam, ceftriaxone, piperacillin/tazobactam, ertapenem, and meropenem). This contrasts with household practices where 9.5% of the population in the community study self-medicated with antibiotics within 30 days of enrollment and reported mostly oral amoxicillin or tetracycline consumption that either do not have the enzymes or mechanisms to effectively select directly for ESCrE or CRE phenotypes or do not reach sufficient concentration in the gut to impact colonization.

Although it is logical to assume that higher ward density, more days spent at the hospital, and intubation are risks for health care transmission of colonizing organisms via gaps in IPC practices, other mechanisms might be involved. For example, these variables may reflect increased physiological stress or significant changes in diet, which are risk factors for altering the gut microbiome [37]. If such changes favor proliferation of Enterobacterales within the gut and if these bacteria are more likely to harbor cephalosporin and carbapenem resistance traits than other taxa [38,39], then non-transmission factors may play an important role. If so, in addition to strengthening IPC to prevent direct transmission, other non-transmission–focused interventions, including antibiotic stewardship programs and measures designed to maintain the microbiome during long-term stays, might help reduce the proliferation of ESCrE and CRE bacteria in hospital settings. More study is needed to differentiate these potential mechanisms, including metagenomic studies that can assess the role of the microbiome in harboring antibiotic resistance genes [40] and direct measures of changes in the abundance and characteristics of Enterobacterales strains because conventional 16S recombinant DNA and metagenomic assays are less likely to detect these relatively less abundant taxa [41].

### Limitations and future studies

Some limitations of this study highlight potential future areas of research. Robust causal inferences can only be identified using longitudinal study designs, including for community and household-based factors that may influence colonization rates before hospital admission and should be considered for future study designs as opposed to the cross-sectional study design used here. As argued previously, the identification of hospital, household, and community risk factors for MDR will benefit from studies that focus on colonization rather than infection alone. Relative to infection-based studies, the potential study population is larger for colonization studies, which allows a more robust sampling frame to systematically evaluate prevalence that is representative of a broader range of participant characteristics. Consequently, studies designed independently from pathological incidence (i.e. infection) or prevalence can generate more power to detect important associations and potential mitigation strategies. This becomes increasingly important in communities where transmission is occurring continually but remains undetected if surveillance is limited to sample collection from patients with infection. Owing to these benefits, colonization studies are also better positioned to routinely monitor the impacts of IPC and antibiotic stewardship efforts in hospitals and communities.

## Conclusion

The results of the current study show that colonization with ESCrE and CRE are associated with antibiotic use and hospital-based factors that are likely to impact bacterial transmission, including hospital stay length, intubation history, and patient density within hospital wards. These results are consistent with previous studies of antibiotic resistance risk factors in hospitals, which have primarily focused on infection but, unlike this study, usually do not control for a patient's domestic environment. Documenting this similarity is important because changes in colonization status during hospitalization can be used consistently to detect the impacts of interventions given that changes in the dynamics of health care–associated infections typically involve longer periods of time and/or larger patient populations to assess efficacy. Design of intervention strategies will benefit from larger-scale studies that provide more accurate assessments of the factors affecting antibiotic resistance in hospitals and by exploring how a patient's colonization status may impact the introduction of antibiotic resistance within hospitals.

## Declarations of competing interest

The authors have no competing interests to declare.

## Appendix Supplementary materials

Image, application 1
