# Supplementary material for: Risk factors for colonization with extended-spectrum cephalosporin-resistant and carbapenem-resistant Enterobacterales among hospitalized patients in Guatemala: An Antibiotic Resistance in Communities and Hospitals (ARCH) study
Source: IJID Reg. 2024 Mar 30;11:100361. doi: 10.1016/j.ijregi.2024.100361 (PMC11021947; doi:10.1016/j.ijregi.2024.100361)
Supplement: Supplementary file 1 [file mmc1.docx]

**Supplement:**

**Risk factors for colonization with extended-spectrum cephalosporin-resistant and carbapenem-resistant Enterobacterales among hospitalized patients in Guatemala: An Antibiotic Resistance in Communities and Hospitals (ARCH) study**

Mark A. Caudell^1^, Carmen Castillo^2^, Lucas F. Santos^2^, Laura Grajeda^2^, Juan Carlos Romero^2^, Maria Renee Lopez^2^, Sylvia Omulo^2,3^, Guy H. Palmer^1^, Douglas R. Call^1^, Celia Cordon-Rosales^1,2,*^, Rachel M. Smith^4^, Carolyn T. A. Herzig^4^, Ashley Styczynski^4^, Brooke M. Ramay^1,2,*,**^

^1^Paul G. Allen School for Global Health, Washington State University, Pullman, WA, USA, ^2^Center for Health Studies, Universidad del Valle de Guatemala, Guatemala City,

^3^Washington State University Global Health-Kenya, Nairobi, Kenya

^4^Division of Healthcare Quality Promotion, U.S. Centers for Disease Control and Prevention, Atlanta, USA,

^x^Central America Regional Office, U.S. Centers for Disease Control and Prevention, Guatemala City, Guatemala

**I. Control variables included in Lasso estimation.**

**Table S1. Category, description, and descriptive statistics of LASSO categorical control variables.**

| **Category** | **Variable description** | **Freq.**  **Yes** | **%**  **Yes** | **Freq.**  **No** | **%**  **No** | **n** |
| --- | --- | --- | --- | --- | --- | --- |
| Animals | Any family member has animals (cattle horses, poultry, or other farm animals) | 286 | 45 | 353 | 55 | 639 |
| Animals | Antibiotics administrated to poultry in the last year | 76 | 12 | 565 | 88 | 641 |
| Animals | Rodents (rats) encountered surrounding the household in the previous year | 304 | 47 | 337 | 53 | 641 |
| Animals | Poultry owned = 0 birds | 373 | 58 | 266 | 42 | 639 |
| Animals | Poultry owned = 1to 5 birds | 101 | 16 | 538 | 84 | 639 |
| Animals | Poultry owned = 6 to 10 birds | 70 | 11 | 569 | 89 | 639 |
| Animals | Poultry owned = 11 to 19 birds | 42 | 7 | 597 | 93 | 639 |
| Animals | Poultry owned >20 birds | 53 | 8 | 586 | 92 | 639 |
| Animals | Dog ownership, categorical = No dogs | 297 | 46 | 343 | 54 | 640 |
| Animals | Dog ownership, categorical = 1 dog | 174 | 27 | 466 | 73 | 640 |
| Animals | Dog ownership, categorical = 2 dogs | 108 | 17 | 532 | 83 | 640 |
| Animals | Dog ownership, categorical ≥ 3 dogs | 61 | 10 | 579 | 91 | 640 |
| Diet in hospital | Normal diet | 243 | 38 | 397 | 62 | 640 |
| Diet in hospital | GI soft diet | 129 | 20 | 511 | 80 | 640 |
| Diet in hospital | Low sodium diet | 36 | 6 | 604 | 94 | 640 |
| Diet in hospital | 1,200 calorie diet | 36 | 6 | 604 | 94 | 640 |
| Diet in hospital | Renal diet | 35 | 6 | 605 | 95 | 640 |
| Diet in hospital | Enteral feeding tube | 38 | 6 | 602 | 94 | 640 |
| Diet in hospital | Liquid diet | 66 | 10 | 574 | 90 | 640 |
| Diet in hospital | Other type of diet | 192 | 30 | 448 | 70 | 640 |
| Diet in hospital | Diet not applicable | 166 | 26 | 474 | 74 | 640 |
| Ethnicity and language | Language at home = Spanish | 470 | 73 | 170 | 27 | 640 |
| Ethnicity and language | Language at home = Mam | 58 | 9 | 582 | 91 | 640 |
| Ethnicity and language | Language at home = Quiche | 87 | 14 | 553 | 86 | 640 |
| Ethnicity and language | Ethnicity = Latino | 366 | 57 | 274 | 43 | 640 |
| Ethnicity and language | Ethnicity = Mam | 87 | 14 | 553 | 86 | 640 |
| Ethnicity and language | Ethnicity = Quiche | 163 | 26 | 477 | 75 | 640 |
| Health seeking | Participant visited clinic for medical attention in past month | 137 | 22 | 500 | 79 | 637 |
| Health seeking | Household member worked in hospital or clinic in past year | 35 | 6 | 606 | 95 | 641 |
| Toilet | Toilet location = inside the household | 282 | 44 | 354 | 56 | 636 |
| Toilet | Toilet location_descriptivedes=Outside the patio area of the household | 151 | 24 | 485 | 76 | 636 |
| Toilet | Toilet_location_descriptive=In the patio of the household | 203 | 32 | 433 | 68 | 636 |
| Health seeking | Clinic visited in last month = 0 times | 500 | 79 | 137 | 22 | 637 |
| Health seeking | Clinic visited in last month = 1 time | 84 | 13 | 553 | 87 | 637 |
| Health seeking | Clinic visited in last month ≥2 times | 53 | 8 | 584 | 92 | 637 |
| Household inventory | Radio | 467 | 73 | 173 | 27 | 640 |
| Household inventory | Television | 534 | 83 | 106 | 17 | 640 |
| Household inventory | Cellphone | 601 | 94 | 38 | 6 | 639 |
| Household inventory | Landline | 63 | 10 | 577 | 90 | 640 |
| Household inventory | Refrigerator | 369 | 58 | 271 | 42 | 640 |
| Household inventory | Washing machine | 128 | 20 | 508 | 80 | 636 |
| Household inventory | Microwave | 238 | 37 | 402 | 63 | 640 |
| Household inventory | Computer | 134 | 21 | 506 | 79 | 640 |
| Household inventory | Internet connection | 170 | 27 | 470 | 73 | 640 |
| Household inventory | Family member uses wristwatch | 274 | 43 | 365 | 57 | 639 |
| Household inventory | Bicycle | 272 | 43 | 367 | 57 | 639 |
| Household inventory | Motorcycle | 229 | 36 | 411 | 64 | 640 |
| Household inventory | Car | 191 | 30 | 447 | 70 | 638 |
| Household inventory | Land for agriculture | 268 | 42 | 372 | 58 | 640 |
| Household inventory | Open fire | 105 | 16 | 535 | 84 | 640 |
| Household inventory | Improved stove with chimney | 319 | 50 | 321 | 50 | 640 |
| Household inventory | Gas stove | 398 | 62 | 242 | 38 | 640 |
| Household inventory | Wood as fuel | 425 | 66 | 215 | 34 | 640 |
| Household inventory | Propane gas as fuel | 397 | 62 | 243 | 38 | 640 |
| Household inventory | All main fuel = liquid propane | 320 | 50 | 320 | 50 | 640 |
| Household inventory | All main fuel = wood | 317 | 50 | 323 | 51 | 640 |
| Household inventory | House = borrowed | 34 | 5 | 606 | 95 | 640 |
| Household inventory | House = lives with family member but doesn't pay | 41 | 6 | 599 | 94 | 640 |
| Household inventory | House = owned | 464 | 73 | 176 | 28 | 640 |
| Household inventory | House = rented | 101 | 16 | 539 | 84 | 640 |
| Household inventory | House improvements in the last year | 93 | 15 | 545 | 85 | 638 |
| Household inventory | Paid housekeeper in the last year | 43 | 7 | 595 | 93 | 638 |
| Household inventory | House roof = concrete tile or terrace | 205 | 32 | 435 | 68 | 640 |
| Household inventory | House roof = zinc sheet or other metallic material | 399 | 62 | 241 | 38 | 640 |
| Household inventory | House floor = cement | 294 | 46 | 346 | 54 | 640 |
| Household inventory | House floor = cement brick (mosaics) | 63 | 10 | 577 | 90 | 640 |
| Household inventory | House floor = ceramic | 208 | 33 | 432 | 68 | 640 |
| Household inventory | House floor = sand or soil | 75 | 12 | 565 | 88 | 640 |
| Household inventory | House wall = adobe | 54 | 9 | 583 | 92 | 637 |
| Household inventory | House wall = cement block | 511 | 80 | 126 | 20 | 637 |
| Household inventory | Bedrooms = 1 | 181 | 28 | 457 | 72 | 638 |
| Household inventory | Bedrooms = 2 | 223 | 35 | 415 | 65 | 638 |
| Household inventory | Bedrooms = 3 | 152 | 24 | 486 | 76 | 638 |
| Household inventory | Bedrooms ≥4 | 82 | 13 | 556 | 87 | 638 |
| Interviewer | Interviewer = 2 | 104 | 16 | 536 | 84 | 640 |
| Interviewer | Interviewer = 3 | 146 | 23 | 494 | 77 | 640 |
| Interviewer | Interviewer = 4 | 131 | 21 | 509 | 80 | 640 |
| Interviewer | Interviewer = 5 | 117 | 18 | 523 | 82 | 640 |
| Interviewer | Interviewer = 6 | 56 | 9 | 584 | 91 | 640 |
| Interviewer | Interviewer = 7 | 86 | 13 | 554 | 87 | 640 |
| Lab outcome | Culture performed for infectious pathology | 135 | 21 | 505 | 79 | 640 |
| Operations in hospital | Number of procedures = 0 | 163 | 26 | 477 | 75 | 640 |
| Operations in hospital | Number of procedures = 1 | 292 | 46 | 348 | 54 | 640 |
| Operations in hospital | Number of procedures = 2 | 136 | 21 | 504 | 79 | 640 |
| Operations in hospital | Number of procedures ≥3 | 49 | 8 | 591 | 92 | 640 |
| Previous Antibiotic Use | Person took antibiotics in last month | 43 | 7 | 597 | 93 | 640 |
| Previous Antibiotic Use | Used amoxicillin last 30 days | 300 | 47 | 340 | 53 | 640 |
| Previous Antibiotic Use | Used tetracycline in last 30 days | 167 | 26 | 473 | 74 | 640 |
| State at admittance | Has chronic disease history | 112 | 18 | 525 | 82 | 637 |
| State at admittance | Patient experiencing an infectious disease process at the time of sample collect | 168 | 26 | 472 | 74 | 640 |
| State at admittance | Pneumonia or lower respiratory tract infection at admittance | 55 | 9 | 585 | 91 | 640 |
| State at admittance | Immuno-compromised upon admittance | 130 | 20 | 511 | 80 | 641 |
| State at admittance | Admitted with infectious disease | 160 | 25 | 481 | 75 | 641 |
| WASH:WATER | Drinking water source = water from a mechanical or manual well (curb) | 54 | 8 | 586 | 92 | 640 |
| WASH:WATER | Drinking water source = bottled water | 255 | 40 | 385 | 60 | 640 |
| WASH:WATER | Drinking water source = pipeline water piped into the household | 192 | 30 | 448 | 70 | 640 |
| WASH:WATER | Drinking water source = pipeline water piped into the patio | 121 | 19 | 519 | 81 | 640 |
| WASH:WATER | Water treatment at home | 366 | 57 | 273 | 43 | 639 |
| WASH:WATER | Boil water | 313 | 49 | 328 | 51 | 641 |
| WASH:WATER | Chlorinate water | 34 | 5 | 607 | 95 | 641 |
| WASH:WATER | Water for washing source = water from a mechanical or manual well (curb) | 75 | 12 | 564 | 88 | 639 |
| WASH:WATER | Water for washing source = pipeline water piped into the household | 335 | 52 | 304 | 48 | 639 |
| WASH:WATER | Water for washing source = pipeline water piped into the patio | 209 | 33 | 430 | 67 | 639 |
| WASH:WATER | Main water source = water from a mechanical or manual well (curb) | 75 | 12 | 562 | 88 | 637 |
| WASH:WATER | Main water source = pipeline water piped into the household | 335 | 53 | 302 | 47 | 637 |
| WASH:WATER | Main water source = pipeline water piped into the patio | 209 | 33 | 428 | 67 | 637 |
| WASH:WATER | River near house | 110 | 17 | 526 | 83 | 636 |
| WASH:WATER | When it rains, water pools up around the house for more than a day | 57 | 9 | 581 | 91 | 638 |
| WASH: TOILET | All sanitary services = toilet connected to sewer | 44 | 7 | 596 | 93 | 640 |
| WASH: TOILET | All sanitary services = latrine | 50 | 8 | 590 | 92 | 640 |
| WASH: TOILET | All sanitary services = toilet connected to septic tank | 56 | 9 | 584 | 91 | 640 |
| WASH: TOILET | All sanitary services = toilet connected to sewer | 374 | 58 | 266 | 42 | 640 |
| WASH: TOILET | All sanitary services = ventilated and improved latrine | 60 | 9 | 580 | 91 | 640 |
| WASH: TOILET | Toilet location = house patio | 203 | 32 | 433 | 68 | 636 |
| WASH: TOILET | Toilet location = inside of house | 282 | 44 | 354 | 56 | 636 |
| WASH: TOILET | Toilet location = outside, not patio | 151 | 24 | 485 | 76 | 636 |
| WASH: TOILET | Shared sanitary service with other house | 60 | 9 | 576 | 91 | 636 |
| WASH: GARBAGE | What is done with natural waste = burn | 31 | 5 | 608 | 95 | 639 |
| WASH: GARBAGE | What is done with natural waste = bury | 90 | 14 | 549 | 86 | 639 |
| WASH: GARBAGE | What is done with natural waste = collection service | 245 | 38 | 394 | 62 | 639 |
| WASH: GARBAGE | What is done with natural waste = placed far from home | 103 | 16 | 536 | 84 | 639 |
| WASH: GARBAGE | What is done with natural waste = put on the patio | 33 | 5 | 606 | 95 | 639 |
| WASH: GARBAGE | What is done with natural waste = use as food for animals | 89 | 14 | 550 | 86 | 639 |
| WASH: GARBAGE | What is done with natural waste = use it as organic fertilizer | 39 | 6 | 600 | 94 | 639 |
| WASH: GARBAGE | Non-natural garbage waste = burn | 298 | 47 | 342 | 53 | 640 |
| WASH: GARBAGE | Non-natural garbage waste = collection service | 304 | 48 | 336 | 53 | 640 |
| WASH: GARBAGE | Garbage collected weekly | 307 | 48 | 334 | 52 | 641 |
| Family member activities | Household member worked in hospital or clinic in past year | 35 | 6 | 606 | 95 | 641 |
| Family member activities | Family member admitted to hospital or visited clinic in past year | 106 | 17 | 535 | 84 | 641 |
| Family member activities | Family member worked in a market in the past year | 42 | 7 | 596 | 93 | 638 |
| Family member activities | Family member works outside the community of residence | 166 | 26 | 474 | 74 | 640 |
| Family member activities | Family member works in market/farm/outside community | 201 | 31 | 440 | 69 | 641 |
|  |  | Freq. of women | % of Women | Freq. of Men | % of Men | Obs |
| Socio-demographics | Sex | 313 | 49 | 327 | 51 | 640 |

**Table S2. Category, description, and descriptive statistics of LASSO continuous control variables.**

| **Variable** | **Mean** | **Std. Dev.** | **Min** | **Max** | **Obs** |
| --- | --- | --- | --- | --- | --- |
| Age | 20.95 | 23.53 | 0.00 | 86 | 640 |
| Number of rooms in the house | 4.53 | 2.12 | 1.00 | 19 | 639 |
| Wealth score | 0.02 | 2.62 | -5.88 | 6.21 | 640 |
| Number of animals in house | 7.26 | 11.43 | 0.00 | 101 | 641 |
| Number of antibiotics administered during hospitalization | 1.32 | 1.513 | 0.00 | 9 | 640 |

**II. Definition of invasive procedure**

The definition of invasive procedure was taken from Sian et al [1]. The definition is **“**An invasive procedure is one where purposeful/deliberate access to the body is gained via an incision, percutaneous puncture, where instrumentation is used in addition to the puncture needle, or instrumentation via a natural orifice.  Where invasive procedures also involve the administration of a medicinal product, these could be categorized as being part of an ‘invasive procedure’ when operator skill is required for its administration within the body, that is, when an internal action is performed to administer the product, or the product is administered to a targeted anatomical area”.

**III. Construction of Wealth Score**

The wealth score was calculated using the Demographic Health Survey Wealth Construction Index, a composite measure of a households cumulative living standard using the component scores for each factor (e.g., assets, house construction materials, water access and sanitation) from the 2014-2015 DHS survey. Score construction followed instructions in [2]

**IV. Control variables retained in LASSO estimation for ESCrE**

Estimation results across ten folds. K-fold cross validation randomly splits the data into 10 parts, with 9 used for training and one for testing. Categorical variables are presented first by variable name and then categories in the subsequent row. Tables where the intercept is presented alone (i.e., “_cons”) indicates that no control variables were selected in the Lasso estimation for the indicated variable.

**Table S3. Control variables retained in ESCrE Model 1 across 10 folds. ESCrE outcome variable.**

| Selected variables | 1 | 2 | 3 | 4 | 5 | 6 | 7 | 8 | 9 | 10 |
| --- | --- | --- | --- | --- | --- | --- | --- | --- | --- | --- |
| _cons | x | x | x | x | x | x | x | x | x | x |

**Table S4. Control variables retained in ESCrE Model 1 across 10 folds: Administration of carbapenems.**

| Selected variables | 1 | 2 | 3 | 4 | 5 | 6 | 7 | 8 | 9 | 10 |
| --- | --- | --- | --- | --- | --- | --- | --- | --- | --- | --- |
| _cons | x | x | x | x | x | x | x | x | x | x |

**Table S5. Control variables retained in ESCrE Model 1 across 10 folds: Administration of Ceftriaxone.** Variable names highlighted and selected categories within the variable immediately below.

| Selected variables | 1 | 2 | 3 | 4 | 5 | 6 | 7 | 8 | 9 | 10 |
| --- | --- | --- | --- | --- | --- | --- | --- | --- | --- | --- |
| **Infectious at sampling** |  |  |  |  |  |  |  |  |  |  |
| No | x |  |  |  |  | x |  |  | x |  |
| Yes |  |  | x |  |  |  |  |  |  |  |
| **Admitted with infectious disease** |  |  |  |  |  |  |  |  |  |  |
| No | x |  |  | x | x | x | x | x | x |  |
| **1200 Calorie Diet** |  |  |  |  |  |  |  |  |  |  |
| No |  | x |  | x | x |  |  |  |  |  |
| _cons | x | x | x | x | x | x | x | x | x | x |

**Table S6. Control variables retained in ESCrE Model 1 across 10 folds: Number of other patients in ward at sampling.**

|  | 1 | 2 | 3 | 4 | 5 | 6 | 7 | 8 | 9 | 10 |
| --- | --- | --- | --- | --- | --- | --- | --- | --- | --- | --- |
| _cons | x | x | x | x | x | x | x | x | x | x |

**Table S7. Control variables retained in ESCrE Model 1 across 10 folds: Hospitalized 4 days or more at time of sampling.**

|  | 1 | 2 | 3 | 4 | 5 | 6 | 7 | 8 | 9 | 10 |
| --- | --- | --- | --- | --- | --- | --- | --- | --- | --- | --- |
| _cons | x | x | x | x | x | x | x | x | x | x |

**Table S8. Control variables retained in ESCrE Model 1 across 10 folds: Hospitalized in previous 30 days.**

|  | 1 | 2 | 3 | 4 | 5 | 6 | 7 | 8 | 9 | 10 |
| --- | --- | --- | --- | --- | --- | --- | --- | --- | --- | --- |
| _cons | x | x | x | x | x | x | x | x | x | x |

**Table S9. Control variables retained in ESCrE Model 1 across 10 folds: Intubated during hospitalization.** Variable names highlighted and selected categories within the variable immediately below.

|  | 1 | 2 | 3 | 4 | 5 | 6 | 7 | 8 | 9 | 10 |
| --- | --- | --- | --- | --- | --- | --- | --- | --- | --- | --- |
| **Admitted with infectious disease** |  |  |  |  |  |  |  |  |  |  |
| No | x |  | x | x | x | x | x |  | x |  |
| _cons | x | x | x | x | x | x | x | x | x | x |

**Table S10. Control variables retained in ESCrE Model 1 across 10 folds: One Invasive procedure.**

|  | 1 | 2 | 3 | 4 | 5 | 6 | 7 | 8 | 9 | 10 |
| --- | --- | --- | --- | --- | --- | --- | --- | --- | --- | --- |
| _cons | x | x | x | x | x | x | x | x | x | x |

**Table S11. Control variables retained in ESCrE Model 1 across 10 folds: Two or more invasive procedures.** Variable names highlighted and selected categories within the variable immediately below.

|  | 1 | 2 | 3 | 4 | 5 | 6 | 7 | 8 | 9 | 10 |
| --- | --- | --- | --- | --- | --- | --- | --- | --- | --- | --- |
| **Antibiotics taken in previous 30 days** |  |  |  |  |  |  |  |  |  |  |
| 1 class of antibiotics taken |  | x |  | x | x |  | x | x |  |  |
| **Interviewer** |  |  |  |  |  |  |  |  |  |  |
| interviewer =2 |  |  |  | x |  |  |  |  | x | x |
| **Clinic visits** |  |  |  |  |  |  |  |  |  |  |
| Visited 2 plus time |  |  |  |  |  |  |  |  | x |  |
| _cons | x | x | x | x | x | x | x | x | x | x |

**III. Control variables retained in LASSO estimation for CrE.**

Estimation results across ten folds. K-fold cross validation randomly splits the data into 10 parts, with 9 used for training and one for testing. Categorical variables are presented first by variable name and then categories in the subsequent row. Tables where the intercept is presented alone indicates that no control variables were selected in the lasso estimation for the indicated variable.

**Table S12. Control variables retained in CrE Model 1 across 10 folds. CrE outcome variable**.

| Selected variables | 1 | 2 | 3 | 4 | 5 | 6 | 7 | 8 | 9 | 10 |
| --- | --- | --- | --- | --- | --- | --- | --- | --- | --- | --- |
| _cons | x | x | x | x | x | x | x | x | x | x |

**Table S13. Control variables retained in CrE Model 1 across 10 folds: Administration of carbapenems.**

| Selected variables | 1 | 2 | 3 | 4 | 5 | 6 | 7 | 8 | 9 | 10 |
| --- | --- | --- | --- | --- | --- | --- | --- | --- | --- | --- |
| _cons | x | x | x | x | x | x | x | x | x | x |

**Table S14. Control variables retained in CrE Model 1 across 10 folds: Administration of Ceftriaxone.** Variable names highlighted and selected categories within the variable immediately below.

| Selected variables | 1 | 2 | 3 | 4 | 5 | 6 | 7 | 8 | 9 | 10 |
| --- | --- | --- | --- | --- | --- | --- | --- | --- | --- | --- |
| **Admitted with infectious disease** |  |  |  |  |  |  |  |  |  |  |
| No | x |  |  | x | x | x | x | x | x |  |
| **1200 Calorie Diet** |  |  |  | x |  |  | x |  |  |  |
| No | x |  |  | x |  |  |  |  |  |  |
| _cons | x | x | x | x | x | x | x | x | x | x |

**Table S15. Control variables retained in CrE Model 1 across 10 folds: Number of other patients in ward at sampling.** Variable names highlighted and selected categories within the variable immediately below.

| Selected variables | 1 | 2 | 3 | 4 | 5 | 6 | 7 | 8 | 9 | 10 |
| --- | --- | --- | --- | --- | --- | --- | --- | --- | --- | --- |
| **Renal diet type** |  |  |  |  |  |  |  |  |  |  |
| No |  | x |  |  |  |  |  |  |  | x |
| _cons | x | x | x | x | x | x | x | x | x | x |

**Table S16. Control variables retained in CrE Model 1 across 10 folds: Hospitalized 4 days or more at time of sampling.**

| Selected variables | 1 | 2 | 3 | 4 | 5 | 6 | 7 | 8 | 9 | 10 |
| --- | --- | --- | --- | --- | --- | --- | --- | --- | --- | --- |
| _cons | x | x | x | x | x | x | x | x | x | x |

**Table S17. Control variables retained in CrE Model 1 across 10 folds: Hospitalized in previous 30 days.**

| Selected variables | 1 | 2 | 3 | 4 | 5 | 6 | 7 | 8 | 9 | 10 |
| --- | --- | --- | --- | --- | --- | --- | --- | --- | --- | --- |
| **Interviewer** |  |  |  |  |  |  |  |  |  |  |
| Interview = 4 |  | x |  |  |  |  |  |  |  |  |
| _cons | x | x | x | x | x | x | x | x | x | x |

**Table S18. Control variables retained in CrE Model 1 across 10 folds: Intubated during hospitalization.**

| Selected variables | 1 | 2 | 3 | 4 | 5 | 6 | 7 | 8 | 9 | 10 |
| --- | --- | --- | --- | --- | --- | --- | --- | --- | --- | --- |
| _cons | x | x | x | x | x | x | x | x | x | x |

**Table S19. Control variables retained in CrE Model 1 across 10 folds: One Invasive procedure.**

| Selected variables | 1 | 2 | 3 | 4 | 5 | 6 | 7 | 8 | 9 | 10 |
| --- | --- | --- | --- | --- | --- | --- | --- | --- | --- | --- |
| _cons | x | x | x | x | x | x | x | x | x | x |

**Table S20. Control variables retained in CrE Model 1 across 10 folds: Two or more invasive procedures.**

| Selected variables | 1 | 2 | 3 | 4 | 5 | 6 | 7 | 8 | 9 | 10 |
| --- | --- | --- | --- | --- | --- | --- | --- | --- | --- | --- |
| _cons | x | x | x | x | x | x | x | x | x | x |

**References**

1. Sian Cousins, Natalie S Blencowe, Jane M Blazeby. What is an invasive procedure? A definition to inform study design, evidence synthesis and research tracking. BMJ Open **2019**; 9:e028576.

2. Rutstein SO. Steps to constructing the new DHS Wealth Index. Rockv MD ICF Int **2015**;
